# Prevalence, Incidence, and Mortality of Stroke in the Chinese Island Populations: A Systematic Review

**DOI:** 10.1371/journal.pone.0078629

**Published:** 2013-11-08

**Authors:** Xiaomei Wu, Bo Zhu, Lingyu Fu, Hailong Wang, Bo Zhou, Safeng Zou, Jingpu Shi

**Affiliations:** 1 Department of Clinical Epidemiology and Center of Evidence Based Medicine, Institute of Cardiovascular, The First Hospital of China Medical University, Shenyang, China; 2 Liaoning Academy of Safety Sciences, Shenyang, China; 3 Department of Environmental and Occupational Health, School of Public Health, China Medical University, Taichung, Taiwan; 4 Department of Neurology, Dalian Municipal Central Hospital, Dalian, China; University of Glasgow, United Kingdom

## Abstract

**Background:**

In China, there are 2.5 million new stroke cases each year and 7.5 million stroke survivors. However, stroke incidence in some island populations is obviously lower compared with inland regions, perhaps due to differences in diet and lifestyle. As the lifestyle in China has changed significantly, along with dramatic transformations in social, economic and environmental conditions, such changes have also been seen in island regions. Thus, we analyzed stroke in the Chinese island regions over the past 30 years.

**Methods:**

We conducted a systematic review to identify reliable and comparable epidemiologic evidence about stroke in the Chinese island regions between 1980 and 2013. Two authors independently assessed the eligibility and the quality of the articles and disagreement was resolved by discussion. Owing to the great heterogeneity among individual study estimates, a random-effects or fixed-effects model was used to incorporate the heterogeneity among records into a pooled estimate for age-standardized rates. Age-standardized rates were calculated by the direct method with the 2000 world population if included records provided the necessary information.

**Results:**

During the past three decades, the overall pooled age-standardized prevalence of stroke is 6.17 per 1000 (95% CI 4.56–7.78), an increase from 5.54 per 1000 (95% CI 3.88–7.20) prior to 2000 to 8.34 per 1000 (95% CI 5.98–10.69) after 2000. However, this difference was not found to be statistically significant. The overall pooled age-standardized incidence of stroke is 120.42 per 100,000 person years (95% CI 26.17–214.67). Between 1982 and 2008, the incidence of stroke increased and mortality declined over time.

**Conclusions:**

Effective intervention and specific policy recommendations on stroke prevention should be required, and formulated in a timely fashion to effectively curb the increased trend of stroke in Chinese island regions.

## Introduction

According to WHO criteria, stroke events are defined as “rapidly developing signs of focal (or global) disturbance of cerebral function lasting greater than 24 hours (unless interrupted by surgery or death) with no apparent nonvascular cause.” [1]. Stroke remains one of the most devastating of all neurological conditions, causing an estimated 5.7 million deaths in 2005 [2]. Stroke is also a major cause of long-term disability [3], [4], with an average of 44 million disability-adjusted life-years lost, with devastating emotional and socioeconomic effects on patients, their families, and the health care system.

In China, with a population of 1.4 billion, the annual stroke mortality rate is approximately 1.6 million, or 157 per 100,000, exceeding heart disease as the leading cause of adult death and disability. In addition, China has 2.5 million new stroke cases each year and 7.5 million stroke survivors [5]. There is also a geographical difference of stroke incidence in China. Northeast China has the highest incidence (486 per 100,000 person-years), whereas in southern China, the incidence is significantly lower (136 per 100,000 person-years), with a male to female ratio of 1.3 to 1.5∶1 [6].

The lifestyle in China has changed rapidly, along with dramatic transformations in social, economic, and environmental conditions over the past 30 years. As a result of an aging population, urbanization, and westernization, the main risk factors for stroke have increased substantially [7], [8]. For example, total fat intake increased from 88.1 grams per day in 1983 to 97.4 grams per day in 2002 [9]. The average serum cholesterol level increased by 24% (4.30 mmol/L to 5.33 mmol/L) and the diabetes prevalence increased from 2.8% to 8.6% from 1984 to1999 [10]. The obesity rate in Beijing increased from 10.19% to 10.41% in urban areas and 6.44% to 20.45% in rural regions between 1984 and 1999 [11]. Thus, China now faces similar risk factors for stroke as Western nations: hypertension, diabetes mellitus, hypercholesterolemia, smoking, coronary artery disease, atrial fibrillation, physical inactivity, obesity, among others. The rising incidence and impact of stroke have created serious public health issues in China [12].

A multi-center collaborative epidemiologic study monitored the incidence of stroke in approximately 100,000 residents in each of 14 study populations between 1991 and 1995 using the MONICA procedure [13]. This study demonstrated that the lowest age-standardized average annual incidence was found in Zhoushan island, in both men (59/100,000 person-years) and women (19/100,000 person-years). The lowest and highest rates (men from Hebei miners and women from Beijing residents) were 7 and 14 times, respectively. The stroke incidence rate in the population of Zhoushan island was obviously low compared with other regions. This may be attributed to the characteristics of their diet, such as high fish protein and unsaturated fatty acids, or less contamination on the island, and a comparatively more comfortable pace of life [14]. Because of the lower incidence of stroke in this region, we found it prudent to perform an analysis of stroke in the island regions.

The islands of China are located in eastern Eurasia and the western edge of the Pacific Ocean, distributed across 38 latitudes and 17 longitudes. From north to south, these islands belong to Liaoning, Tianjin, Hebei, Shandong, Jiangsu, Shanghai, Zhejiang, Fujian, Taiwan, Guangdong, Guangxi, Hainan and other coastal provinces (municipalities and autonomous regions). The aims of this systematic review are to provide current knowledge of stroke in Chinese island populations with all available population-based stroke prevalence, incidence, or mortality studies, and to analyze secular trends in stroke. We hope to advance our understanding of stroke frequency and discover the determinants in Chinese island populations, providing evidence for formulating the strategy for the prevention and control of stroke in the Chinese island populations.

## Methods

### Search Strategy

We searched the Chinese Biomedical literature database (CBM), Chinese National Knowledge Infrastructure (CNKI), Medline, Embase, Web of Science, Science Citation Index, and Cochrane Library electronic databases (1980 to 2013). Keywords included in the search were: stroke, stroke disease, cerebral hemorrhage, cerebral ischemia, cerebral infarction, cerebrovascular, island, fishermen, insular, China, and Chinese. For example, search strategy for Medline: “(((((((stroke [Title/Abstract]) OR cerebral hemorrhage [Title/Abstract]) OR cerebral ischemia [Title/Abstract]) OR cerebral infarction [Title/Abstract]) OR cerebrovascular [Title/Abstract])) AND (((((((((((((((((((((island [Title/Abstract]) OR island counties [Title/Abstract]) OR insular areas [Title/Abstract]) OR island populations [Title/Abstract]) OR island residents [Title/Abstract]) OR fishermen [Title/Abstract]) OR island people [Title/Abstract]) OR island masses [Title/Abstract]) OR Changhai [Title/Abstract]) OR Changdao [Title/Abstract]) OR Chongming [Title/Abstract]) OR Zhoushan [Title/Abstract]) OR Shengsi [Title/Abstract]) OR Daishan [Title/Abstract]) OR Dinghai [Title/Abstract]) OR Putuo [Title/Abstract]) OR Yuhuan [Title/Abstract]) OR Dongtou [Title/Abstract]) OR Pingtan [Title/Abstract]) OR Dongshan [Title/Abstract]) OR Nanao [Title/Abstract])) AND (((("China" [MeSH Terms] OR "China" [All Fields]) OR ("asian continental ancestry group" [MeSH Terms] OR ("asian" [All Fields] AND "continental" [All Fields] AND "ancestry" [All Fields] AND "group" [All Fields]) OR "asian continental ancestry group" [All Fields] OR "Chinese" [All Fields]))).” In our search strategy for Medline, the words “Changhai,” “Changdao,” “Chongming,” “Zhoushan,” “Shengsi,” “Daishan,” “Dinghai,” “Putuo,” “Yuhuan,” “Dongtou,” “Pingtan,” “Dongshan,” and “Nanao,” names of administrative counties in the Chinese islands, were used in order to avoid missing reports that did not contain the search terms “island,” “fishermen,” “insular areas,” and others.

**Figure 1:**
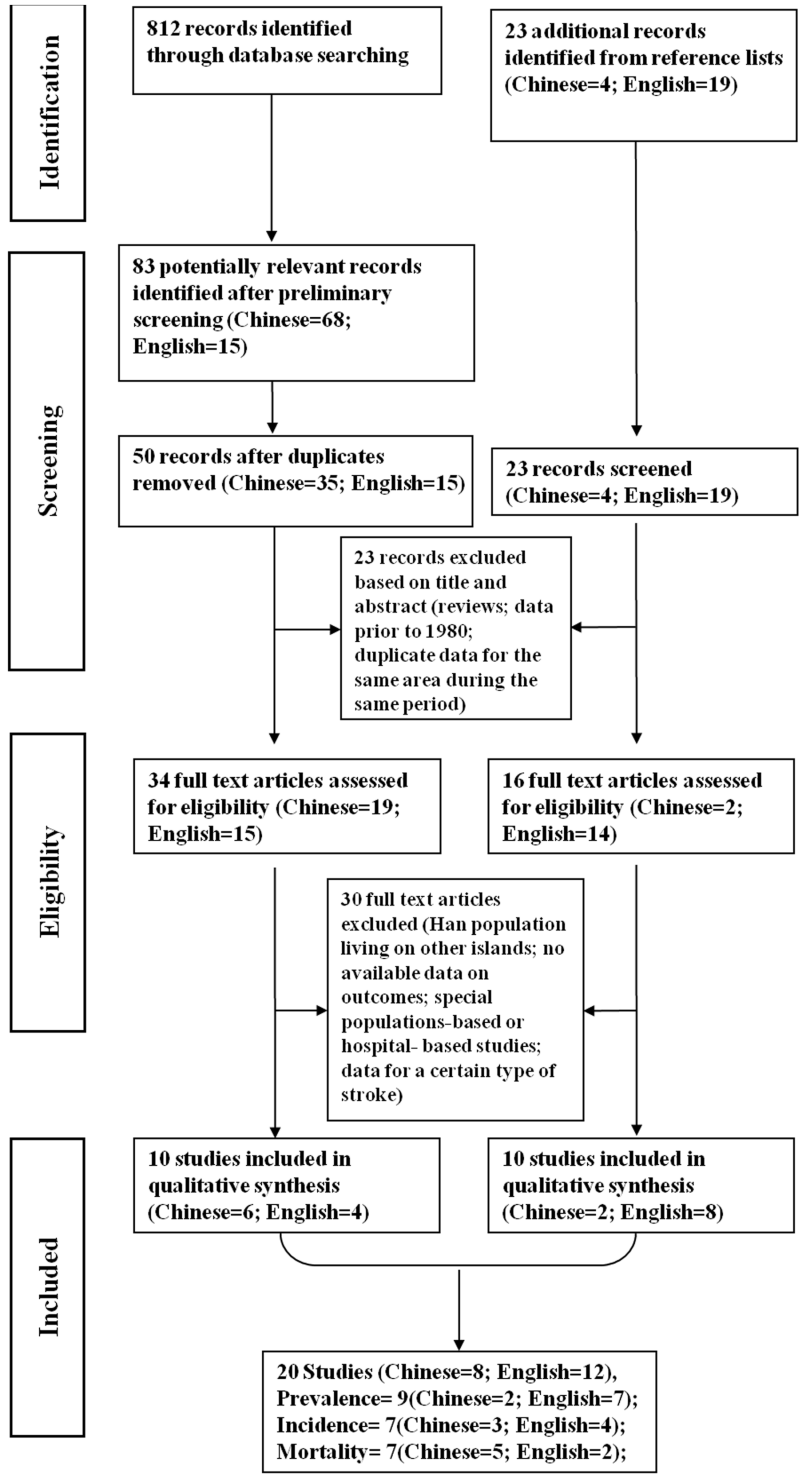
Flow diagram (selection strategy) of included studies.

To optimize our data search, we also scanned reference lists of each original and review article identified for relevant studies, in addition to reviews and meta-analyses, and consulted with key experts in the field. The search was restricted to articles published in English or Chinese. Identified publications were reviewed by one of the authors (Wu XM).We identified 23 additional records from this process (Figure 1).

### Study Selection

Two authors (Wu XM and Zhu B) independently assessed articles and abstracts for eligibility, and any disagreements were resolved by discussion. In this review, we refer to a record as any unique report from the published, grey, or unpublished literature. If several records were reported from the same population during overlapping periods, we included the record, providing the longest time span for analysis.

Records had to fulfill the following criteria to be eligible for inclusion in our review: (1) research conducted wholly or partially in Chinese island populations; (2) clear diagnostic criteria for stroke; (3) adult population-based study; (4) original article published from January 1980 to January 2013; and (5) published in English or Chinese.

Exclusion criteria were as follows: (1) Han population living on non-Chinese islands; (2) special population-based or hospital-based studies; (3) the original article did not involve the available data for outcomes; (4) repeated report; (5) reviews, letters, and comments; (6) low-quality article; (7) confined to only one pathologic type of stroke; and (8) results presented only as abstracts. We identified 20 records through this process that provided relevant prevalence, incidence, or mortality data. Different subsets of records were potentially eligible for different parts of this review (Figure 1).

### Data Extraction

Data extraction was managed in Microsoft Excel. Records meeting inclusion criteria were formally reviewed by pairs of reviewers, using a data extraction form based on previous reviews. Our definition included records with sufficient data to derive an estimate, even if a rate had not been explicitly reported in the original records. Derived rates were calculated and rechecked (Wu XM and Zhu B). Data abstracted for each record were confirmed by reviewer consensus.

For each record that fulfilled criteria, we extracted the following information: first author’s name, title, publication year, publication source (or unpublished), publication reference, object age (mean or range), object gender, case-finding methodology, case-finding duration (years), geographic region, diagnostic outcomes (stroke, ischemic stroke, or hemorrhagic stroke), method of outcome assessment (prevalence, incidence, or mortality rate), diagnostic criteria, number of events, sample size, and adjustment type (crude, adjusted or standardized).

### Quality Assessment

Table 1, which contained 7 items, was utilized to evaluate the quality of the records. Two authors assessed all records. A study was considered low quality if it did not meet more than 2 items. Disagreements between reviewers were resolved by discussion.

**Table 1:** Quality assessment of the individual studies.

first author	1	2	3	4	5	6	7
Pan BJ [19]	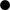	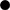	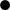	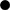	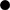	○	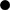
Wang DX [20]	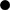	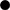	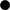	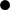	○	○	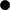
Hu HH [21]	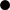	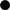	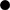	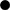	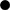	○	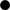
Hu HH [22]	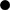	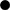	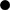	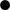	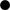	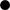	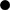
Hu HH [23]	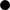	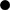	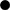	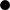	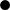	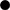	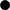
Su CL [24]	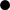	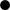	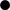	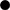	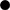	○	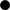
Lee TK [25]	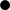	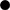	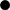	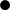	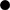	○	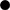
Fuh JL [26]	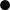	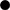	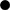	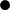	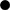	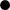	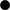
Fuh JL [27]	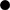	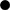	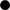	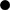	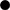	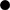	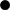
Huang ZS [28]	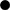	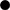	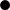	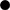	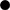	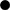	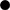
Zhou BF [13]	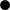	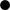	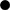	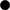	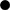	○	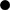
Lee YT [30]	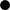	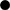	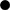	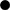	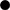	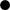	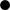
Liu CG [31]	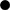	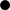	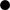	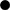	○	○	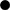
Wang JF [14]	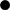	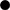	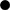	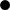	○	○	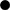
Lin HC [32]	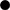	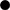	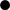	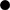	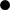	○	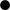
Zou SY [34]	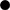	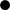	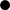	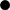	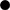	○	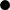
Song SZ [35]	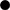	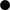	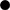	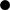	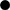	○	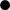
Miu S [36]	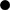	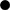	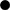	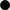	○	○	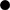
Ye Z [37]	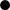	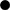	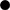	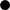	○	○	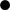
Hu GZ [38]	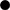	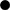	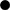	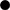	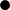	○	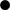

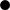
: The item is met.

○: The item is not met.

1. Describe the locations.

2. Describe periods of recruitment, follow-up, and data collection.

3. Give the eligibility criteria, and the sources and methods of selection of participants.

4. Involve the available data on outcomes.

5. Report numbers of individuals at each stage of study.

6. Give reasons for non-participation at each stage.

7. Give characteristics of study participants.

### Data Analysis and Statistical Methods

Owing to the great heterogeneity among individual study estimates, which could be due to genuine regional differences and wide age ranges, age-standardized rates were calculated by the direct method with 2000 world population data, if included records provided the necessary information. Based on these reasons, the random-effects or fixed-effects model attempts to incorporate heterogeneity among records into a pooled estimate only for age-standardized rates [15].

Extracted data were analyzed using Stata (version 12.0) software. To calculate the pooled age-standardized rates of prevalence or incidence, we used the random-effects model, with the standard method proposed by Dersimonian, and the Laird [16] or fixed-effects model using an inverse variance statistical method [17]. In each meta-analysis, χ^2^ and I^2^ values were first calculated to assess the heterogeneity of the included records. When there was no statistically significant heterogeneity, the pooled effect was calculated using a fixed-effects model; otherwise, a random-effects model was employed.

## Results

### Flow of Included Studies

A total of 835 records were identified by the search strategy, 815 of which were excluded (Figure 1). 20 records met inclusion criteria, 18 were published articles, and the remaining 2 were graduate theses. Of all 20 records, the prevalence, incidence, and mortality analyses were included in 9, 7, and 7 records, respectively (Figure 1).

### Study Characteristics

Table 1 presents the quality of individual studies; Table 2 presents the characteristics (summary and results) of individual studies. For stroke mortality rate, we were more concerned about whole population data, as most of the included records also did. Therefore a record (Gao CW [18]) about mortality rate of stroke in age-specific population was excluded for the above reason.

**Table 2:** Characteristics of records and patients included in the review.

first author	publication year	age(range)	gender	case-finding duration(years)	geographical region	outcome	sample size	crude rate	adjustmentrate #	diagnostic criteria
Pan BJ [19]	1984	40–	both	1982	Taiwan Province	prevalence	NR	14.0	4.83	NR
Wang DX [20]	1986	35–59	both	1982	Putuo County, Zhejiang Province	prevalence	1590	6.3	1.87	WHO
Hu HH [21]	1986	all	Ma/Fe	1972–1983	Taiwan Province	mortality	NR	88.0/72.5	NR	ICD-9
Hu HH [22]	1989	36–	both	1986.10.1–12.3	Taiwan Province	prevalence	8705	16.4	5.76	WHO
Hu HH [23]	1992	36–	both	1986.10–1990.10	Taiwan Province	incidence	31502	330	136.41	WHO
Su CL [24]	1992	all	Ma/Fe	1989	Taiwan Province	mortality	no	76.6/67.7	no	no
Lee TK [25]	1995	65–	both	1990–1992	Taiwan Province	prevalence	2600	59.6	5.77	WHO
Fuh JL [26]	1996	50–	both	1993.8.1–1994.9.17	Kinmen, Taiwan Province	prevalence	3915	24.5	4.87	WHO
Fuh JL [27]	2000	50–	both	1993.8–1997.12	Kinmen, Taiwan Province	incidence	10057	527	105.12	WHO
Huang ZS [28]	1997	all	both	1994.10–12	Taiwan Province	prevalence	11925	5.95	6.56	Other [29]
Zhou BF [13]	1998	25–74	both	1991–1995	Zhoushan City, Zhejiang Province	incidence	255380	31.33	16.96	WHO
Lee YT [30]	2000	35–	both	1990	Taipei, Taiwan Province	prevalence	3602	21.9	9.12	Other†
Lee YT [30]	2000	35–	both	1990–1995	Taipei, Taiwan Province	incidence	12542	590	245.76	Other†
Liu CG [31]	2002	all	both	1982–2001	Putuo County, Zhejiang Province	mortality	2011492	12.88	NR	ICD-9
Liu CG [31]	2002	all	both	1982–2001	Putuo County, Zhejiang Province	incidence	2011492	20.73	NR	WHO
Wang JF [14]	2007	all	both	1998–2006	Daishan County, Zhejiang Province	incidence	495000	95.85	NR	Other‡
Lin HC [32]	2007	35–	both	2001.8–2002.1	Taiwan Province	prevalence	9794	19.3	9.21	Other [33]
Zou SY [34]	2008	all	both	2000–2004	LongIsland County, Shandong Province	mortality	86371	152.83	NR	ICD-10
Song SZ [35]	2008	all	both	2002–2005	Xiangshan County, Zhejiang Province	mortality	2107534	128.54	NR	ICD-10
Miu S [36]	2010	all	both	2005–2008	Chongming County, Shanghai	mortality	NR	229	NR	ICD-10
Miu S [36]	2010	all	both	2005–2008	Chongming County, Shanghai	incidence	NR	268	NR	Other‡
Ye Z [37]	2011	25–	both	2009	Xiangshan County, Zhejiang Province	prevalence	2313	11.7	6.69	NR
Hu GZ [38]	2011	all	both	1986–1988	Daishan County, Zhejiang Province	mortality	644448	74.17	NR	ICD-9
Hu GZ [38]	2011	all	both	2009–2010	Daishan County, Zhejiang Province	mortality	382464	136.22	NR	ICD-10

Unless otherwise stated, all prevalence sample sizes and rates are expressed as persons and per 1000 population; all incidence and mortality sample sizes and rates are expressed as person-years and per 100,000 person-years.

#: Adjust to the WHO (2000) world population calculated from available data.

NR: not reported and not calculated from available data.

† : a history of hemiparesis or hemiplegia confirmed by neurologists from National Taiwan University Hospital (NTUH) by physical examination.

‡ : 1995 Fourth National cerebrovascular disease Conference.

### Prevalence of Stroke in Chinese Island Populations

During the three decades, data relating to stroke prevalence containing at least 44,444 persons, from 9 records were available for analysis. The total crude stroke prevalence rates ranged from 5.95 to 59.6 per 1000; the lowest crude prevalence was noted by Huang [28] (1994, age range: all), and the highest crude prevalence was observed by Lee [25] (1992, age range: more than 65) (Table 2).

**Figure 2:**
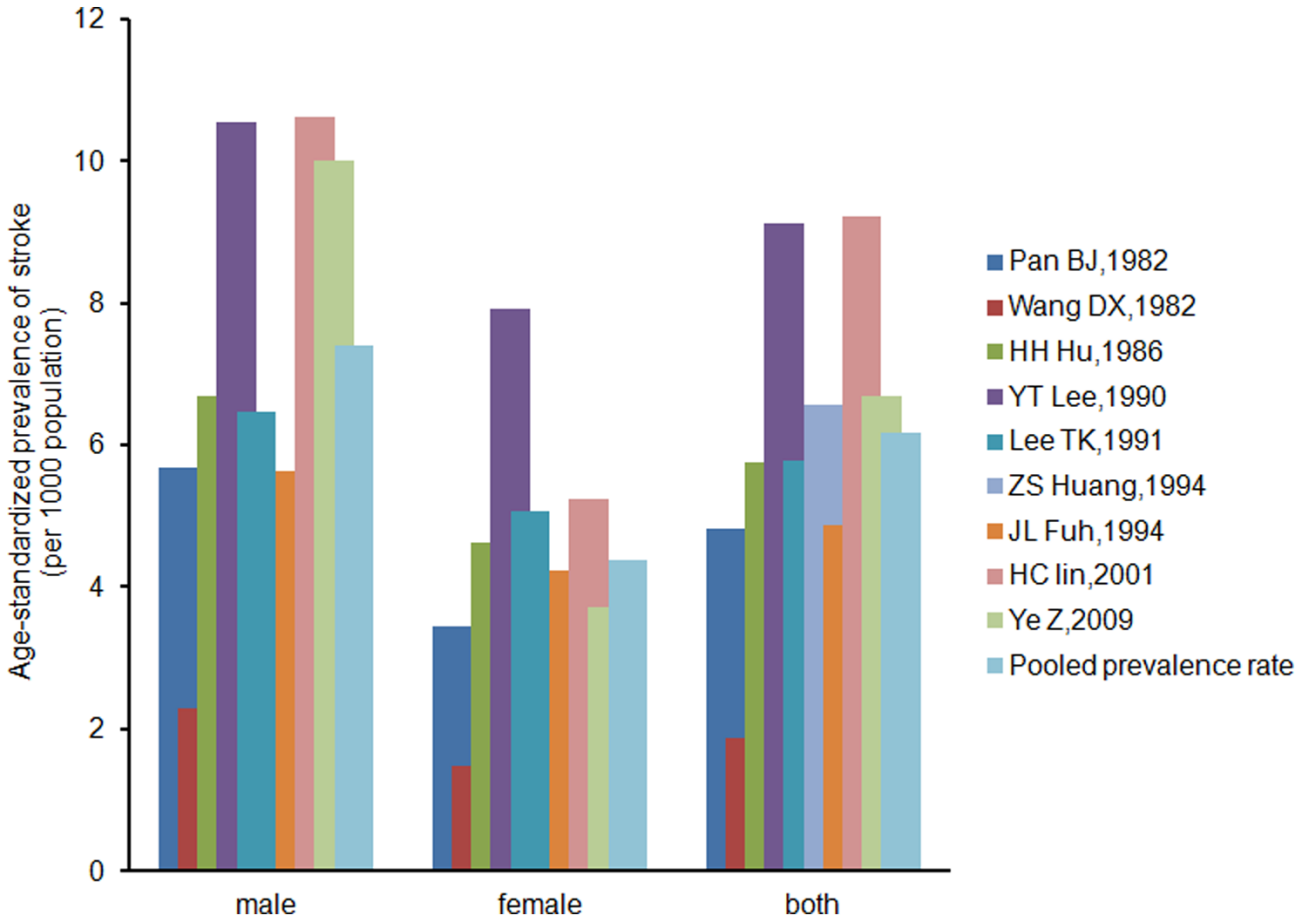
Age-standardized prevalence of stroke per 1000 in selected studies (adjusted to the WHO world population). Studies are arranged in ascending order of research time.

Age-standardized prevalence rates in 9 records ranged from 1.87 per 1000 (Wang [20], 1982) to 9.21 per 1000 (Lin [31], 2001) (Figure 2). The results of the meta-analysis showed that the overall pooled prevalence of stroke was 6.17 per 1000 (95% CI 4.56–7.78, p for heterogeneity = 0.000, I^2^ = 77.6%), as determined using the random-effects model; the male pooled stroke prevalence was 7.40 per 1000 (95% CI 4.43–10.37, p for heterogeneity = 0.004, I^2^ = 71.6%), as determined by the random-effects model; the female pooled stroke prevalence was 4.37 per 1000 (95% CI 3.18–5.55, p for heterogeneity = 0.131, I^2^ = 41.2%), as determined by the fixed-effects model.

**Figure 3:**
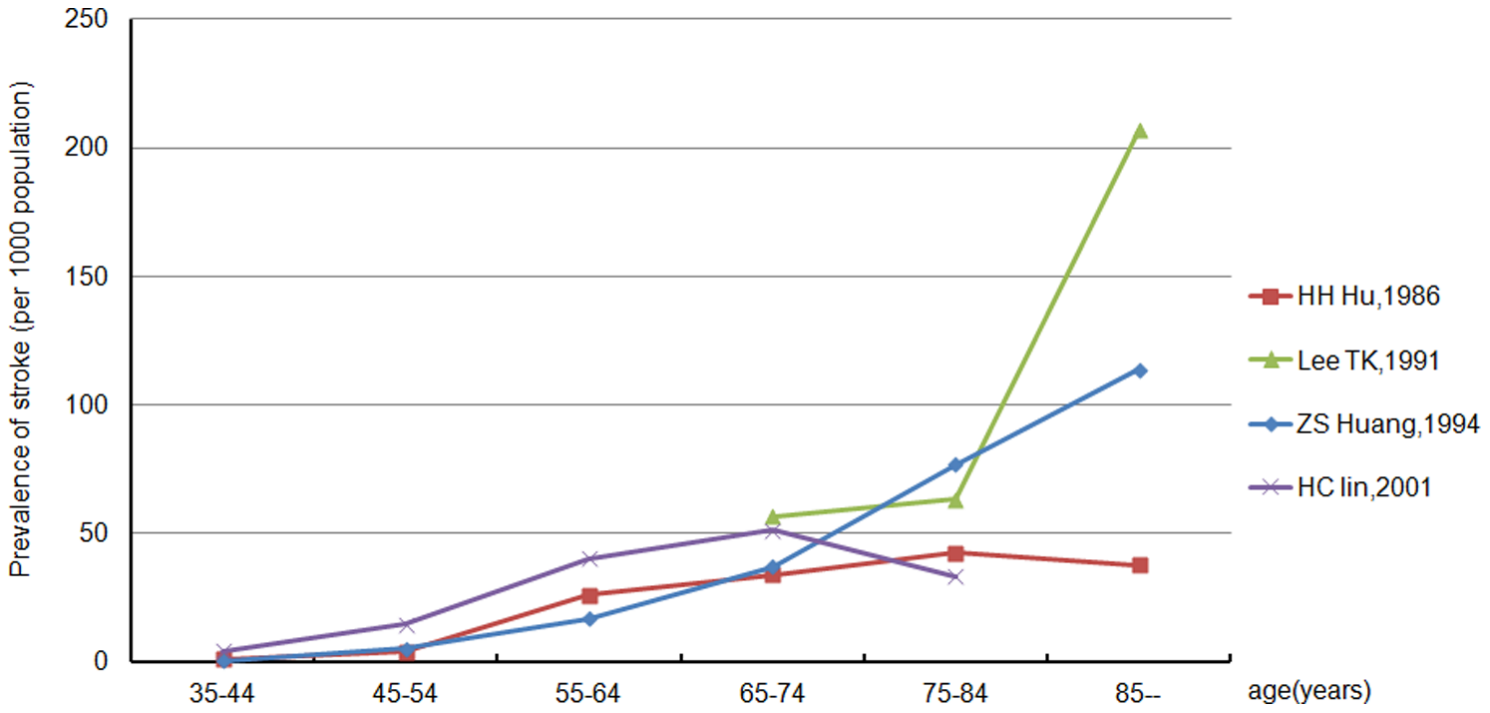
Graph showing the prevalence of stroke by different ages in selected studies. Studies are arranged in ascending order of research time.

The age-specific stroke prevalence was reported in 4 records (Hu [22], Lee [25], Huang [28], Lin [31]). In general, data indicated that stroke prevalence increased with age. But according to Hu [22] and Lin [31], stroke prevalence began to decline in the oldest age group (Figure 3).

**Figure 4:**
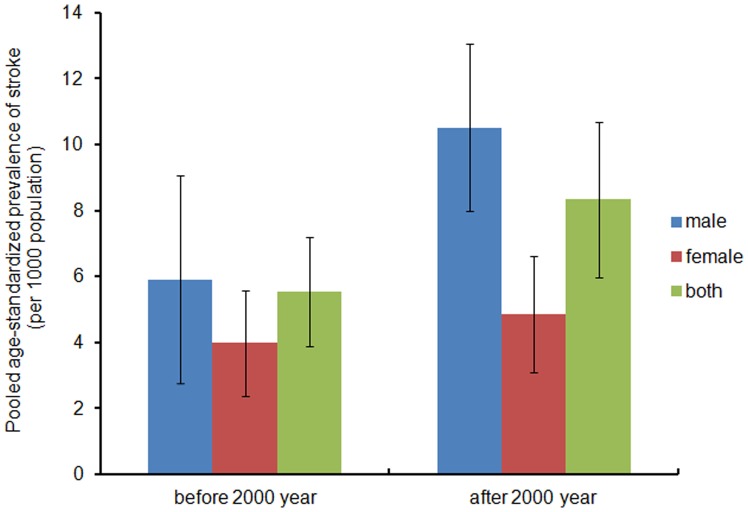
Pooled age-standardized prevalence of stroke (per 1000) in selected studies by different periods.

The pooled estimates of age-standardized stroke prevalence rate was 5.54 per 1000 (95% CI 3.88–7.20) prior to 2000 and 8.34 per 1000 (95% CI 5.98–10.69) after 2000; in males, the pooled age-standardized prevalence was 5.91(95% CI 2.76–9.06) prior to 2000 and 10.51(95% CI 7.97–13.05) after 2000; in females, it was 3.98 (95% CI 2.38–5.58) and 4.84 (95% CI 3.07–6.61). The values of pooled age-standardized prevalence increased, especially in the male population. However, this change was not found to be statistically significant, possibly as a result of fewer reports (Figure 4).

### Incidence of Stroke in Chinese Island Populations

Data relating to stroke incidence containing at least 2,815,973 cumulative person years from 7 records were available for analysis. During the study period, a total of 1155 stroke patients were found (not including patients from Miu [36], because they were not reported in his thesis). The average annual crude incidence (per 100,000 person years) ranged from 20.73 to 590.0, with the lowest incidence reported by Liu [31](1982–2001, age range: all) and the highest by Lee [30] (1990–1995, age range: more than 35) (Table 2).

**Figure 5:**
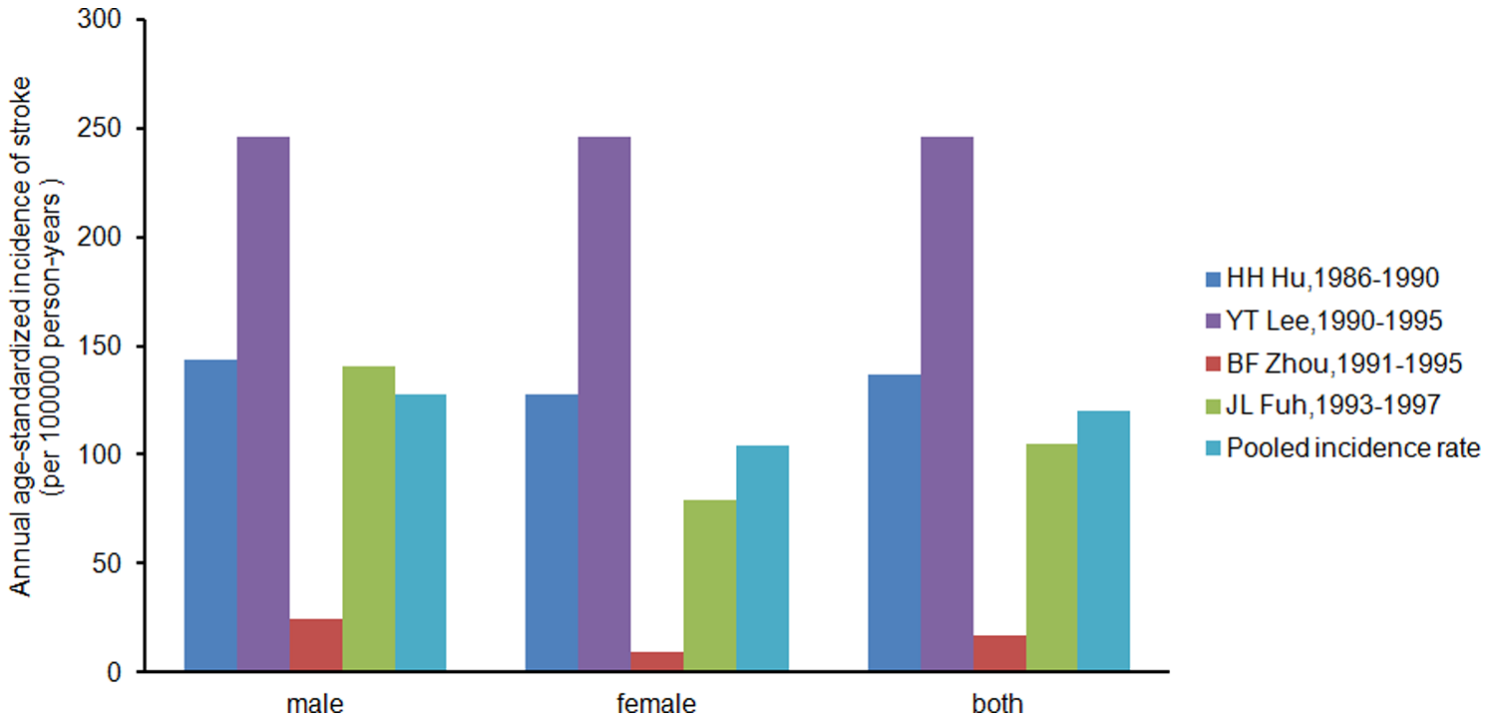
Annual age-standardized incidence of stroke per 100,000 person-years in selected studies (adjusted to the WHO world population). Studies are arranged in ascending order of research.

Average annual age-standardized incidence rates (per 100,000 person years) among 4 of 7 records (not calculated from available data in Liu [31], Wang [14], and Miu [36]) ranged from 16.96 (Zhou [13], 1991–1995) to 245.76 (Lee [30], 1990–1995). The meta-analysis revealed the heterogeneity of the 4 included studies (I^2^ = 95.4%, p for heterogeneity = 0.000). Analysis using a random-effects model revealed an overall pooled incidence of 120.42 (95% CI 26.17–214.67); for males 127.96 (95% CI 29.07–226.85, I^2^ = 90.5%, p for heterogeneity = 0.000) and for females 104.36 (95% CI 11.64–197.09, I^2^ = 91.4%, p for heterogeneity = 0.000) (Figure 5).

**Figure 6:**
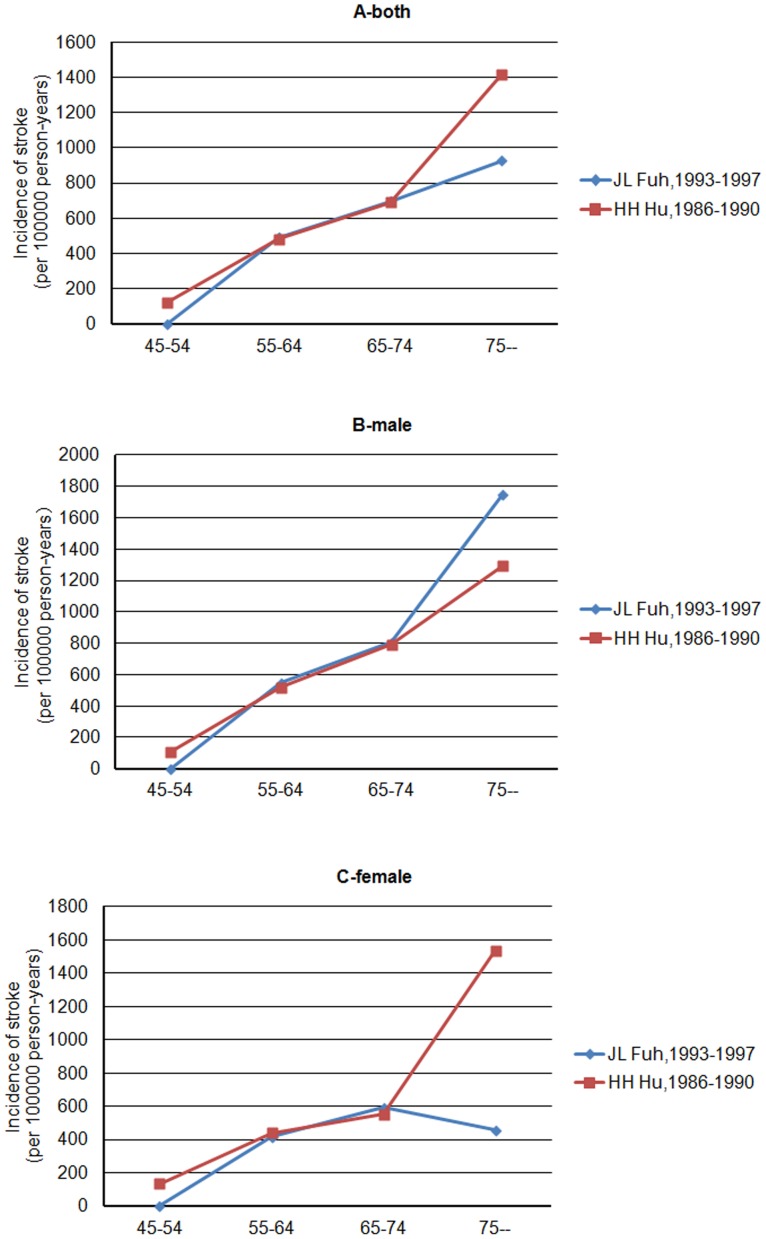
Graph showing the incidence of stroke by different ages in selected studies, for both (panel A) and men or women (panel B or C).

The age-specific incidence of stroke was reported only from 2 records (Hu [23], Fuh [27]) and both data indicated that stroke incidence increased with age. But according to Fuh [27], stroke incidence in females began to decline in patients older than 75 years (Figure 6).

We identified 3 primary records from 7 studies that directly investigated possible changes in the crude stroke incidence over time in Chinese island populations, ranging from 20 year (Liu [31], 1982–2001), 9 years (Wang [14],1998–2006) and 4 years (Miu [36], 2005–2008), respectively.

**Figure 7:**
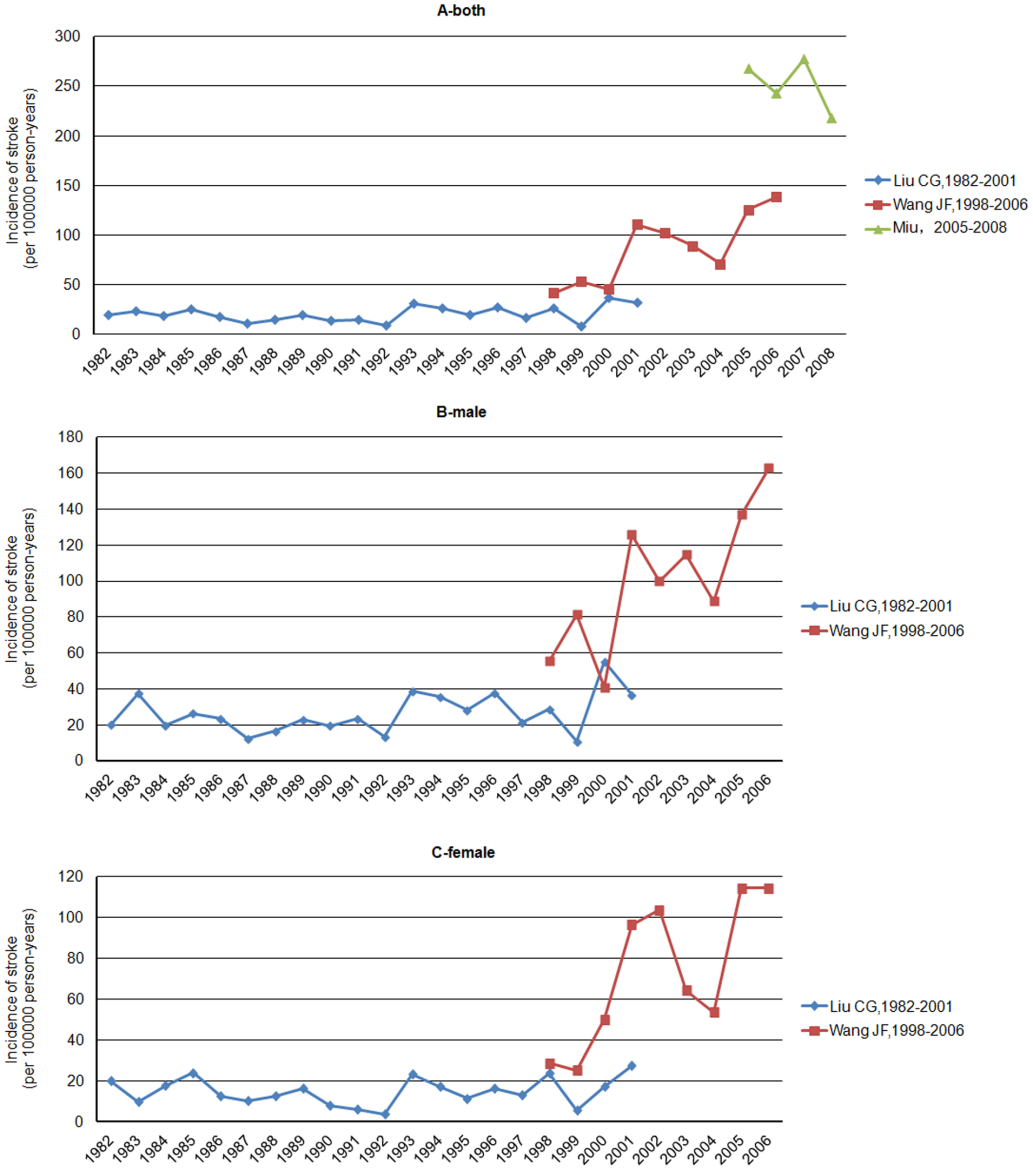
Graphs showing the secular trend of annual crude incidence of stroke in selected studies included objects of all ages, for both (panel A) and men or women (panel B or C).

Wang [14] reported an increase in stroke incidence between 1998 and 2006 (from 41.8 to 138.2 per 100,000 person years), at the same rates for males and females. Liu(1982–2001) [31] and Miu(2005–2008) [36] found a slight increase in stroke incidence over time. As a whole, these three records (1982–2008) demonstrated an increase of stroke incidence with time (Figure 7).

### Mortality of Stroke in Chinese Island Populations

**Figure 8:**
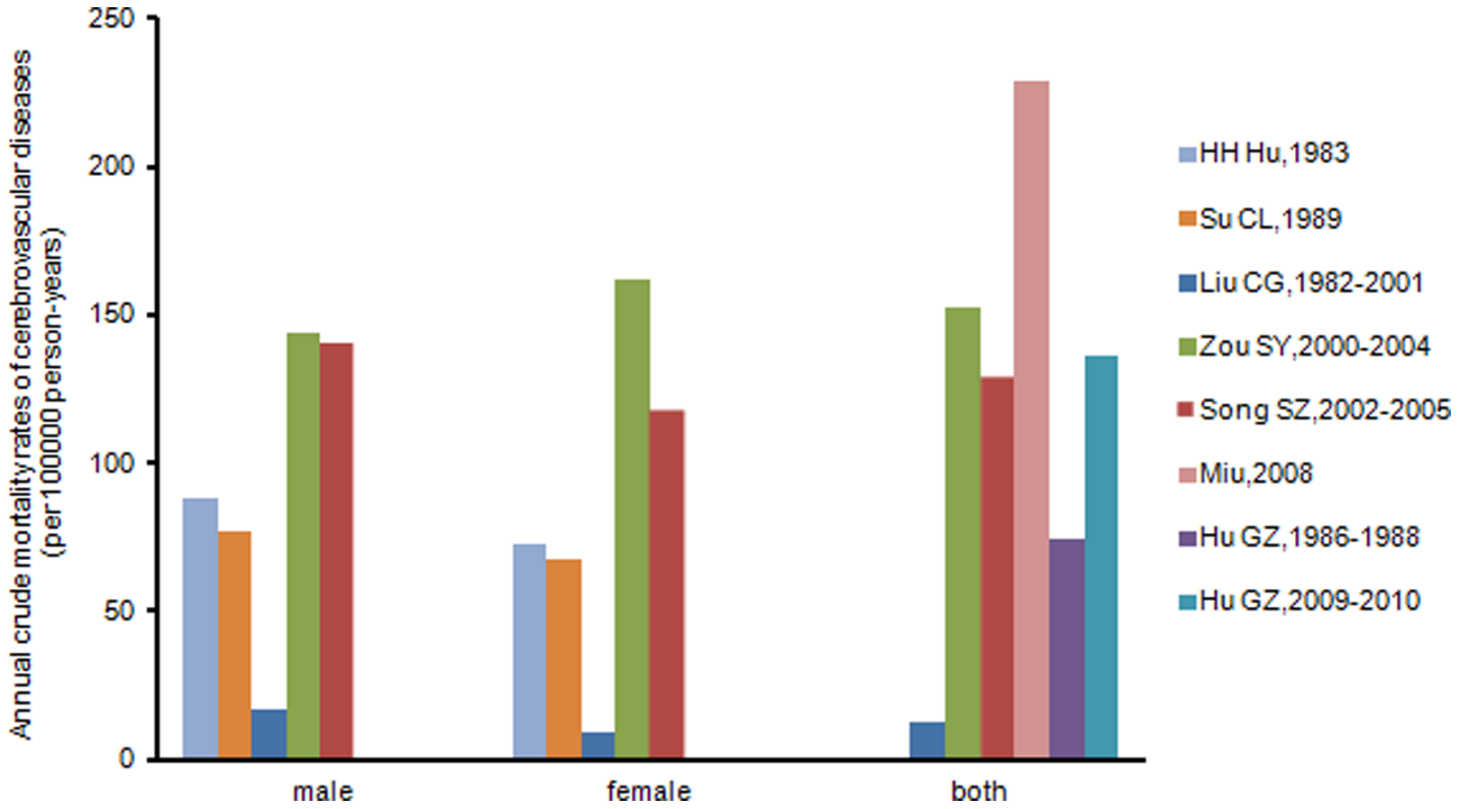
Annual crude mortality rates of cerebrovascular diseases per 100,000 person-years in selected studies.

Between 1982–2010, the lowest crude mortality rate of stroke was reported by Liu [31], between 1982–2001 (12.88 per 100,000 person years) and the highest reported by Miu [36] in 2008 (229 per 100,000 person years) (Table 2, Figure 8).

Records evaluating secular trends in annual crude stroke mortality rate in Chinese island populations were available only for Liu [31] (1982–2001), Song [35] (2002–2005), Miu [36] (2005–2008), and Hu [21] (male and female respectively, 1980–1983).

Over the three decades, the crude stroke mortality rates in the target population of Liu [31] decreased by 72.21% (from 17.47 in 1982 to 4.85 in 2001, per 100,000 person-years); the crude mortality rates of Song [35] declined by 10.49% (from140.31 in 2002 to 125.59 in 2005, per 100,000 person-years). There is no clear secular trend displayed in data from Miu [36]. However, during the earlier period (1980–1983), a similar increase for both males and females was observed by Hu [21].

**Figure 9:**
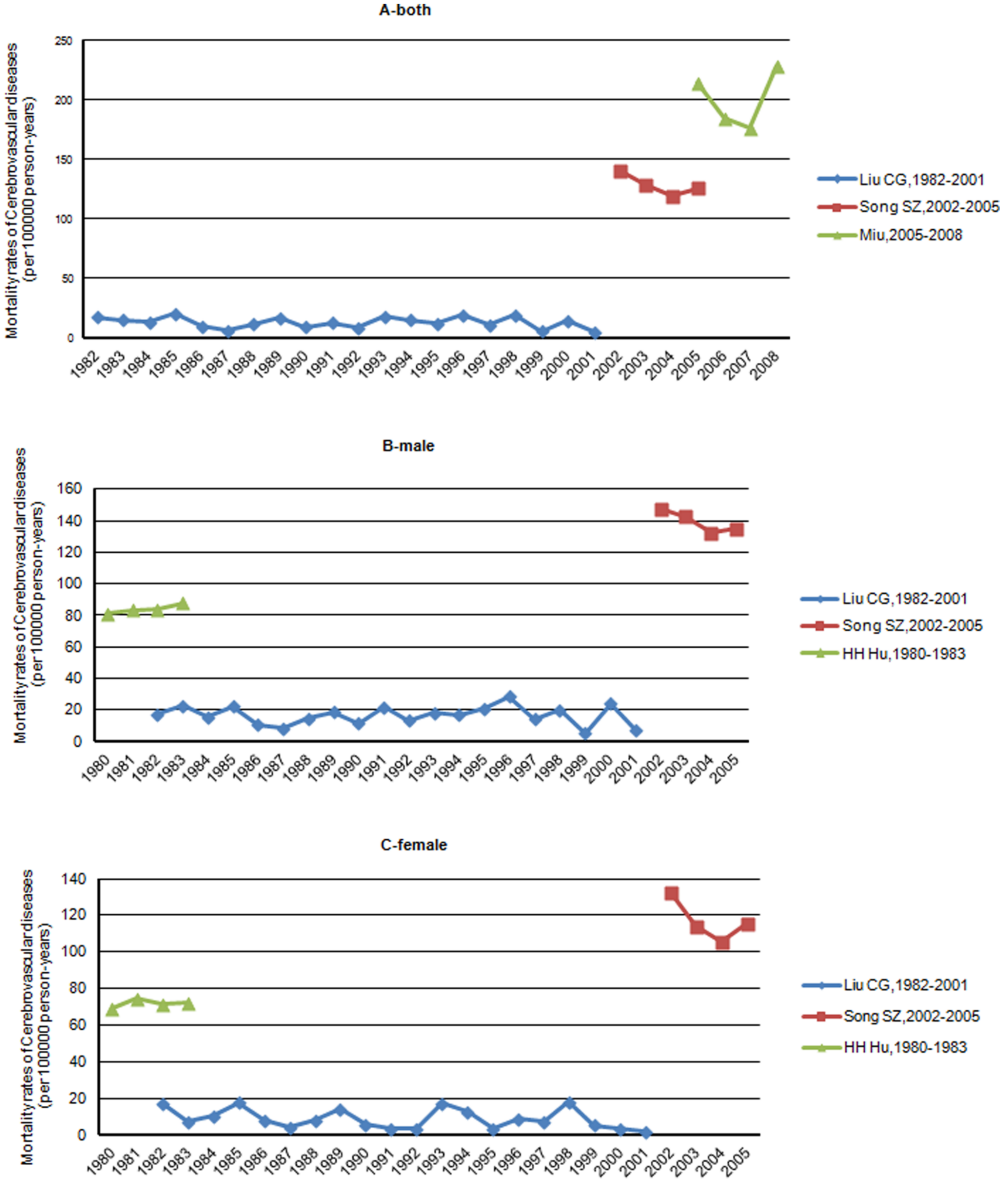
Graphs showing the secular trend of annual crude mortality rates of cerebrovascular diseases in selected studies included objects of all ages, for both (panel A) and men or women (panel B or C).

Overall, the crude stroke mortality rate is declining with time, although mortality rates in different records increased over time (12.88, Liu [31]1982–2001; 128.54, Song [35]2002–2005; 229, Miu [36]2005–2008) because of the heterogeneity between studies, from regional or research methodological differences(Figure 9).

## Discussion

To the best of our knowledge, this is the first systematic review to provide pooled estimates on stroke in Chinese island populations. Stroke incidence rates in Chinese island populations increased over the past three decades (Figure 7), at an even faster rate than in surrounding areas [14]. This is probably due to improvements in health care systems and diagnostic ability [8], [12]; however, more likely due to health and epidemiological transition [9], [39], [40]. Currently, there are no adequate analyses for the causes of the increased incidence of stroke in Chinese island populations. More research on this issue is required.

An increased frequency of stroke-related risk factors, such as hypertension, diabetes mellitus, body-mass index, hyperlipidemia, smoking, and alcoholism[41]–[44], are due to nutrition and lifestyle changes in Chinese island populations over the past thirty years [45]. First, transportation and exchanges between the island and mainland has improved in recent years, leading to decreased intake of marine fish food and increased intake of poultry and other food [14]. Second, a habitual high-salt diet[46]–[50] (salted or pickled fish) and low intake of fresh fruits and vegetables [51], [52] are still very common in island residents, which has been corrected through education in the mainland population [40]. Third, due to changes in industrial structure, the number of island residents engaged in manual labor reduced although with the same labor intensity [40]. Fourth, with an aging population, increased life expectancy or unplanned urbanization or air pollution brought by industry development plays an environmental role[40], [53]–[55].

Hypertension as an independent risk factor for stroke has been surveyed in several recent trials[56]–[58]. According to the World Health Organization, 62% of all strokes are attributable to high blood pressure [59]. The age-standardized prevalence of hypertension among island residents of Zhoushan City (Zhang [60]) increased from 5.28% to 14.96%, or 183.33% overall between 1990 and 2003.

Abnormal glucose regulation, including diabetes, impaired fasting glucose, and impaired glucose tolerance, have been recognized as important risk factors for occurrence and recurrence of ischemic stroke in Europe and America[61]–[63]. The prevalence of diabetes in China is now approaching that of the United States (7%) [6]. The age-standardized prevalence of diabetes in the population of Zhoushan island had been 12.5% in men and 7.2% in women (1998) [64].

The mean serum total cholesterol (TC) level of 160.7 mg/dl for men and 158.5 mg/dl for women was lower in the population of Zhoushan island compared to other populations between 1992–1994 [13]. However the age-standardized prevalence of high serum TC increased substantially from 7.6% to 23.0% in men and from 10.4% to 23.6% in women between 1992 and 1998, close to the average level [64].

The age-standardized prevalence of overweight in the population of Zhoushan island increased from 6.2% to 17.1% in men and 7.3% to 23.6% in women, 175.81% and 223.29% overall respectively, between 1982 and 1998 [64]. This finding will undoubtedly lead to an increased prevalence and incidence of stroke.

Alcohol and tobacco use are increasingly common in China. With the exception of Mongolia, smoking rates are higher in Chinese men than in any other country. The age-standardized prevalence of alcohol use was 71.0% and tobacco was 67.4% in men of Zhoushan island in1998 [64].

Most risk factors for stroke appeared in the Chinese island populations during the last 30 years, and approximated the nationwide average. However, our findings regarding stroke incidence and mortality do not provide evidence that changes in stroke mortality correlate with stroke incidence. In actuality, WHO MONICA project data [65] suggests that changes in stroke mortality are largely attributable to early fatality rather than increasing incidence in most populations. With economic development and medical progress fatality [9] and long-term morbidity has decreased [3], [66], [67]. Therefore, Chinese island populations may have benefited from access to advanced treatment, lowering mortality rates (Figure 9; Liu [31], Song [35]). However, the burden of stroke has increased with the cumulative effect of risk factors for stroke [12]. Effective preventive measures will not only improve health, but will contribute to sustainable economic development in the Chinese islands [40], [68].

The prevalence and incidence of stroke in one record in this review is higher in men (Figure 2, 5), similar to most studies. This may be due to differences in risk factors such as hypertension, diabetes, hyperlipidemia, and alcohol and tobacco use [69].

Figures 3 and 6 show data on age-specific prevalence and incidence, which rose exponentially with age; this was not unexpected, since the strongest risk factor for stroke is age. The prevalence rate, however, fell in the oldest age group (Hu [22], Lin [31]) which may be due to smaller sample size, increasing the uncertainty around prevalence, or because of the higher mortality rates in the highest age group.

The strengths of this study are four-fold. First, we used population-based stroke studies on prevalence, incidence, or mortality. 20 included studies ensured that the data provided was accurate. Second, the study period was comprised of the past three decades, a long period. Third, in addition to descriptive analyses, we used meta-analytic techniques, providing pooled estimates [15] over different study periods, thus increasing statistical reliability. Fourth, we provided a detailed account of our search strategy and data extraction methodology, which allowed us to identify all citations relevant to our review.

Our review has several limitations. There was still not enough data for pooled estimates of population-based stroke mortality and some subgroup analyses (stroke subtypes). Second, there was heterogeneity on stroke incidence and prevalence in the included records; we used a random effects model to incorporate this statistical heterogeneity, which we were unable to explain. The heterogeneity may have come from the complex interplay between genetics, vascular risk factors, and health seeking behavior, which must also be highlighted as a potential weakness.

## Supporting Information

Checklist S1 **PRISMA Checklist of this systematic review.**
(DOC)
