# Correction: Prevalence, Incidence, and Mortality of Stroke in the Chinese Island Populations: A Systematic Review

**DOI:** 10.1371/annotation/7db95d69-ea24-4580-b0b1-20e37788df8e

**Published:** 2014-01-02

**Authors:** Xiaomei Wu, Bo Zhu, Lingyu Fu, Hailong Wang, Bo Zhou, Safeng Zou, Jingpu Shi

Affiliation number 3 for the second author is incorrect. The correct affiliation number 3 is: Department of Environmental and Occupational Health, School of Public Health, China Medical University, Shenyang China. 

